# *Klebsiella pneumoniae* Carbapenemase Producers in South Korea between 2013 and 2015

**DOI:** 10.3389/fmicb.2018.00056

**Published:** 2018-01-25

**Authors:** Eun-Jeong Yoon, Jung Ok Kim, Dokyun Kim, Hyukmin Lee, Ji Woo Yang, Kwang Jun Lee, Seok Hoon Jeong

**Affiliations:** ^1^Department of Laboratory Medicine, Yonsei University College of Medicine, Seoul, South Korea; ^2^Centers for Disease Control and Prevention, National Institute of Health, Cheongju, South Korea

**Keywords:** KPC, Tn*4401*, carbapenem resistance, *Klebsiella pneumoniae* ST307, carbapenemase producing *Enterobacteriaceae*

## Abstract

Between 2014 and 2015, the *Klebsiella pneumoniae* carbapenemase (KPC) was becoming endemic in South Korea. To assess this period of transition, we analyzed KPC producers in terms of molecular epidemiology. A total of 362 KPC-producing *Enterobacteriaceae* strains, including one from 2013, 13 from 2014, and 348 from 2015, were actively collected from 60 hospitals throughout the peninsula. Subtypes of KPC were determined by PCR and direct sequencing, and isotypes of Tn*4401* (the transposon flanking the *bla*_KPC_ gene) were specified by PCR using isotype-specific primers and direct sequencing. Sporadic occurrence of KPC-producing *Enterobacteriaceae* was initially observed around Seoul, which is the most crowded district of the country, and these strains rapidly disseminated in 2014, to the other parts of the country in 2015. The bacterial clones responsible for the extreme epidemiological transition were *K. pneumoniae* ST307 (46.2%) and ST11 (21.3%). Less frequently, *E. coli* (4.7%), *Enterobacter* spp. (1.4%), and other *Enterobacteriaceae* members (1.7%) producing the enzyme were identified. The *bla*_KPC-2_ gene bracketed by Tn*4401a* (72.1%) was the most prevalent mobile genetic element responsible for the dissemination, and the same gene carried either by Tn*4401b* (2.2%) or Tn*4401c* (6.6%) was identified at a lesser frequency. The genes *bla*_KPC-3_ (1.6%) and *bla*_KPC-4_ (6.4%), both flanked by Tn*4401b*, were occasionally identified. This study showed endemic dissemination of KPC producers in 2015 due to a clonal spread of two *K. pneumoniae* strains. Further systemic surveillance is needed to monitor dissemination of KPC-producers.

## Introduction

Carbapenemases often jeopardize chemotherapy for infectious diseases caused by common nosocomial pathogens. Currently, *Klebsiella pneumoniae* carbapenemases (KPC) belonging to the Ambler class A carbapenemases are the most clinically worrisome enzymes because of their global spreads and a broad-spectrum of substrates including most of the β-lactam drugs, except cephamycins (Munoz-Price et al., 2013; Pitout et al., 2015). KPC producers were first identified for *K. pneumoniae* from the United States in 1996 (Yigit et al., 2001) and, so far, a total of 23 KPC variants have been identified (http://www.lahey.org/Studies). KPC producers, particularly among *Enterobacteriaceae* isolates, are now being extensively reported worldwide. In some regions of the world, such as North America (some regions of the USA), Latin America (Colombia, Brazil, and Argentina), Europe (Greece and Italy), the Middle East (Israel), and Asia (some regions of China), KPC dissemination has reached endemic levels (Albiger et al., 2015; Mehta et al., 2015).

The rapid dissemination of the *bla*_KPC_ gene is known to be derived from the clonal spread of *K. pneumoniae* sequence type 258 (ST258) (Munoz-Price et al., 2013; Pitout et al., 2015). Besides, horizontal gene transfer drives KPC dissemination via a Tn*3*-type active transposon Tn*4401* enclosing the *bla*_KPC_ gene. The transposon is bracketed by 39-bp imperfect inverted repeat sequences capable of mobilizing the *bla*_KPC_ gene at high frequency and it generates a 5-bp target duplication by insertion without target site specificity (Cuzon et al., 2011). At present, nine isotypes of Tn*4401* (Tn*4401a* to Tn*4401i*) differing by the upstream sequences of the *bla*_KPC_ gene were identified. The *bla*_KPC_ gene promoters varied by the Tn*4401* isotypes control the level of gene expression resulting in varied levels of carbapenem resistance (Naas et al., 2012; Cheruvanky et al., 2017).

The National Laboratory Surveillance System (NLSS) for CPEs started in November 2010 by the Korea Center for Disease Prevention and Control (KCDC) with 100 sentinel general hospitals (unpublished data). Through the surveillance study, an alarming transition of KPC epidemiological stages to inter-regional spread was observed between 2014 and 2015. The population of KPC producers and the inter-regional movement of core clones needed to be further investigated and, in this study, the molecular epidemiology was assessed with actively collected KPC producers located throughout the peninsula.

## Materials and methods

### Strains

A total of 362 non-duplicate KPC-producing *Enterobacteriaceae* strains (one from 2013, 13 from 2014, and 348 from 2015) were collected voluntarily from 60 hospitals: 16 NLSS-non-participating hospitals including 12 general hospitals, two convalescent hospitals, and two clinics, and 44 NLSS-participating general hospitals. Further testing including the species identification by 16S rDNA sequencing was performed in a reference laboratory.

### Antimicrobial susceptibility and resistance analyses

Antimicrobial susceptibility to eight drugs (aztreonam, cefotaxime, ceftazidime, cefoxitin, gentamicin, amikacin, ciprofloxacin, and tigecycline) was evaluated by the disk diffusion method on Mueller-Hinton (MH) agar (Difco Laboratories, Detroit, MI), following CLSI guidelines (Clinical and Laboratory Standards Institute, 2015b). The MICs for colistin were assessed by the broth microdilution method using MH broth (Difco Laboratories), following the recommendations of the Joint CLSI-EUCAST Polymyxin Breakpoints Working Group (The joint CLSI–EUCAST and Polymyxin Breakpoints Working Group, 2016). The MICs for imipenem and meropenem were determined by the agar dilution method, following CLSI guidelines (Clinical and Laboratory Standards Institute, 2015a). Both *Escherichia coli* ATCC 25922 and *Pseudomonas aeruginosa* ATCC 27853 were used for quality control. The *bla*_KPC_ gene was detected by PCR using gene-specific primers (Supplementary Table), and direct sequencing was carried out for subtyping KPC. Additional resistance determinants were identified by PCR and direct sequencing: *bla*_CTX-*M*-1-like_, *bla*_CTX-*M*-9-like_, and *bla*_CTX-*M*-25-like_ for CTX-M-type extended-spectrum β-lactamases (ESBLs); *bla*_ACT_, *bla*_ACC_, *bla*_CMY_, and *bla*_DHA_ for plasmid-mediated AmpCs; and *armA, rmtA*, and *rmtB* for16S ribosomal methyltransferases (Perez-Perez and Hanson, 2002; Ryoo et al., 2005). Multiple-carbapenemase producers of positive amplification either of *bla*_OXA-48-like_, *bla*_IMP_, *bla*_VIM_, *bla*_GES_, and *bla*_NDM_ genes were eliminated from the study.

### Isotyping the Tn*4401* transposon

To determine the isotypes of Tn*4401*, isotype-specific forward primers for the five most common isotypes (*a* to *e*) (Naas et al., 2012) were newly designed (Supplementary Table) and PCR was carried out with a universal reverse primer targeting the *bla*_KPC_ gene. The amplicons were sequenced for verification.

### Multilocus sequence typing (MLST)

PCR and sequencing were carried out for seven housekeeping genes: *gapA, infB, mdh, pgi, phoE, rpoB*, and *tonB* for *K. pneumoniae* (Diancourt et al., 2005), and *adk, fumC, gyrB, icd, mdh, purA*, and *recA* for *E. coli* (Wirth et al., 2006). The nucleotide sequences obtained for both DNA strands were compared with sequences in the MLST database for each species (http://bigsdb.web.pasteur.fr/klebsiella for *K. pneumoniae* and http://mlst.warwick.ac.uk/mlst/dbs/Ecoli for *E. coli*), to determine allelic numbers and STs.

## Results

### The epidemiology of KPC-producing *Enterobacteriaceae*

Among the 362 KPC-producing *Enterobacteriaceae* strains, *K. pneumoniae* (334/362, 92.2%) was the predominant bacterial species and *E. coli* (17/362, 4.7%) was followed by *Enterobacter* spp. (5/362, 1.4%), *Citrobacter* spp. (3/362, 0.8%), *Klebsiella oxytoca* (1/362, 0.3%), *Serratia marcescens* (1/362, 0.3%), and *Hafnia alvei* (1/362, 0.3%) were identified less frequently.

Three subtypes KPC-2 (334/362, 92.3%), KPC-3 (6/362, 1.7%), and KPC-4 (22/362, 6.1%) were identified. Major isotype of the Tn*4401* bracketing the *bla*_KPC_ gene was Tn*4401a* (261/362, 72.0%) and, less frequently, Tn*4401b* (36/362, 9.9%) and Tn*4401c* (24/362, 6.6%) were identified. Tn*4401b* was linked with all three subtypes of the *bla*_KPC_ gene, while Tn*4401a* and Tn*4401c* carried only the *bla*_KPC-2_ gene. For the non-isotypable 41 transposons (11.3%) by the Tn*4401*-isotyping PCR, amplification between the 3′-end of the upstream IS*Kpn7* and the *bla*_KPC_ gene was tried and only one of the 41 was identified having a truncated Tn*3* at the isotype-determining region, while none produced any amplicons. The non-isotype-determined (ND) Tn*4401*s were coupled only with *bla*_KPC-2_ gene, whether in *K. pneumoniae* (*n* = 38) or in *E. coli* (*n* = 3).

The *bla*_KPC-2_ gene bracketed by Tn*4401a* (Tn*4401a*[*bla*_KPC-2_]) was harbored by *K. pneumoniae* (*n* = 243) of varied STs [mainly ST307 (*n* = 155), ST11 (*n* = 37), and ST789 (*n* = 21)], *E. coli* (*n* = 11) [mostly of ST1624 (*n* = 7)], and other *Enterobacteriaceae* [*Enterobacter* spp. (*n* = 2), *C. freundii* (*n* = 2), *C. koseri* (*n* = 1), *K. oxytoca* (*n* = 1), and *S. marcescens* (*n* = 1)] (Table 1). Tn*4401b*[*bla*_KPC-2_] was carried either by *K. pneumoniae* (*n* = 7), five ST11 and two ST307, or by *Enterobacter* spp. (*n* = 1). Tn*4401c*[*bla*_KPC-2_] was carried only by *K. pneumoniae* (*n* = 24) belonging to ST307 (*n* = 8), ST789 (*n* = 6), ST11 (*n* = 5), ST48 (*n* = 3), ST392 (*n* = 1), and ST2230 (*n* = 1). The Tn*4401b*[*bla*_KPC-3_] was identified in *E. coli* ST393 (*n* = 2) and in *K. pneumoniae* (*n* = 4, specifically two ST314 and one each of ST15 and ST697) and Tn*4401b*[*bla*_KPC-4_] was identified in *K. pneumoniae* (specifically five ST392, four ST101, three ST11, two ST307, and one of each ST4, ST48, ST54, and ST627), *E. coli* ST2847 (*n* = 1), *Enterobacter* spp. (*n* = 2), and *Hafnia alvei* (*n* = 1).

**Table 1:** The *bla*_KPC_ mobile elements carried by KPC producers in South Korea.

	***E. coli*** **belonging to ST**									***K. pneumoniae*** **belonging to ST**																								***Enterobacter* spp**.	***C. freundii***	***C. koseri***	***K. oxytoca***	***S. marcescens***	***H. alvei***	**Total**
	**1642**	**131**	**2448**	**3306**	**4683**	**1193**	**410**	**393**	**2847**	**307**	**11**	**789**	**48**	**392**	**290**	**101**	**2538**	**29**	**314**	**524**	**628**	**15**	**1035**	**1393**	**1413**	**147**	**17**	**2230**	**317**	**697**	**4**	**54**	**627**							
**2013**																																								
*KPC-2*																												*1*												*1*
Tn*4401c*																												1												1
**2014**																																								
*KPC-2*	*7*									*2*	*1*			*1*																										*11*
Tn*4401a*	7									2																														9
Tn*4401c*														1																										1
ND											1																													1
*KPC-3*																						*1*																		*1*
Tn*4401b*																						1																		1
KPC-4														1																										1
Tn*4401b*														1																										1
**2015**																																								
*KPC-2*		*2*	*1*	*1*	*1*	*1*	*1*			*163*	*73*	*34*	*13*	*1*	*6*	*1*	*3*	*2*		*2*	*2*		*1*	*1*	*1*	*1*	*1*		*1*					*3*	*2*	*1*	*1*	*1*		*321*
Tn*4401a*		1	1	1	1					153	37	21	10	1	6	1	3	2		1			1	1	1	1	1							2	2	1	1	1		251
Tn*4401b*										2	5																							1						8
Tn*4401c*										8	5	6	3																											22
ND		1				1	1				26	7								1	2								1											40
*KPC-3*								*2*											*2*											*1*										*5*
Tn*4401b*								2											2											1										5
*KPC-4*									*1*	*2*	*3*		*1*	*4*		*5*															*1*	*1*	*1*	*2*					*1*	*22*
Tn*4401b*									1	2	3		1	4		5															1	1	1	2					1	22
Total	7	2	1	1	1	1	1	2	1	167	77	34	14	7	6	6	3	2	2	2	2	1	1	1	1	1	1	1	1	1	1	1	1	5	2	1	1	1	1	362

*Numbers in italics, sum of the sub-titled values*.

### Dissemination of KPC producers throughout the South Korean Peninsula from 2013 to 2015

One *K. pneumoniae* isolate ST2230/Tn*4401*c[*bla*_KPC-2_] was collected in Seoul in 2013. In 2014, 13 KPC producers were identified including six *K. pneumoniae* strains, i.e., two ST307/Tn*4401*a[*bla*_KPC-2_], one ST11/ND[*bla*_KPC-2_], one ST392/Tn*4401c*[*bla*_KPC-2_], one ST392/Tn*4401b*[*bla*_KPC-4_], and one ST15/Tn*4401b*[*bla*_KPC-3_], along with seven *E. coli* ST1642/Tn*4401*a[*bla*_KPC-2_] strains from a single-hospital outbreak in a suburb of Seoul (Incheon). In 2015, a total of 348 KPC producers were identified including 327 *K. pneumoniae* [mainly ST307/Tn*4401*a[*bla*_KPC-2_] (153/327, 46.8%), ST11/Tn*4401*a[*bla*_KPC-2_] (37/327, 11.3%), ST25/ND[*bla*_KPC-2_] (25/327, 7.6%), and ST789/Tn*4401*a[*bla*_KPC-2_] (21/327, 6.4%)] and 10 *E. coli* strains of nine subclones. Notably, five KPC-producing *K. pneumoniae* isolates were collected in 2015 from other healthcare facilities than general hospitals: ST307/Tn*4401*a[*bla*_KPC-2_], ST11/Tn*4401*a[*bla*_KPC-2_], and ST290/Tn*4401*a[*bla*_KPC-2_]) from two convalescent hospitals and ST48/Tn*4401*c[*bla*_KPC-2_] and ST307/Tn*4401*a[*bla*_KPC-2_] from two clinics.

**Figure 1:**
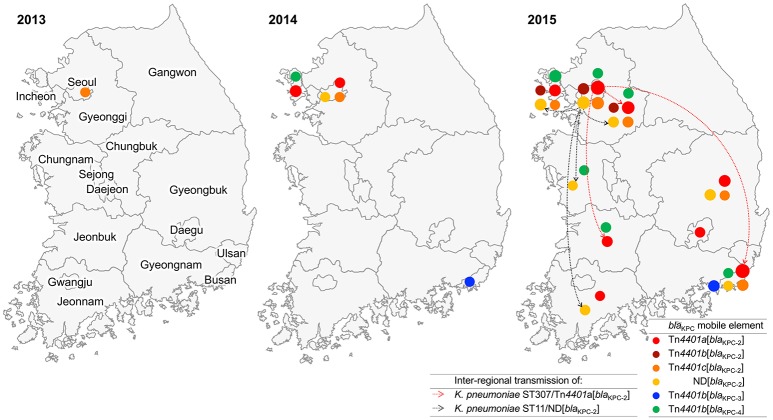
Appearance and inter-regional dissemination of KPC producers carrying varied Tn*4401* isotypes between 2013 and 2015. Colored dots indicate the subtypes of the *bla*_KPC_ gene and isotypes of Tn*4401* that were located in each district. The size of each dot represents the number of isolates on a log scale. Inter-regional dissemination of the two KPC-producing clones is denoted with broken arrows.

The *K. pneumoniae* ST307/Tn*4401*a[*bla*_KPC-2_], ST11/ND[*bla*_KPC-2_], and ST392/Tn*4401b*[*bla*_KPC-4_] clones were identified over two consecutive years. In 2014, two *K. pneumoniae* ST307/Tn*4401*a[*bla*_KPC-2_] were first identified in Seoul. In 2015, the same clone was identified with 153 strains in Seoul (*n* = 49) and three more districts including a suburb of Seoul (Gyeonggi, *n* = 12), a southeastern peninsula (Busan, *n* = 91), and a southwestern peninsula (Jeonbuk, *n* = 1; Figure 1). Besides, the *K. pneumoniae* ST11/ND[*bla*_KPC-2_] clone was first identified in Seoul in 2014 and, in 2015, the same clone was identified in Seoul (*n* = 12), two suburbs of Seoul (Gyeonggi, *n* = 1; Incheon, *n* = 1), and in southeastern peninsula (Gyeongbuk, *n* = 2; Busan, *n* = 1). The *K. pneumoniae* ST392/Tn*4401b*[*bla*_KPC-4_] clone from Incheon in 2014 was subsequently identified with four isolates in 2015 from the same district.

### Carbapenem resistance conferred by the *bla*_KPC_ gene

**Figure 2:**
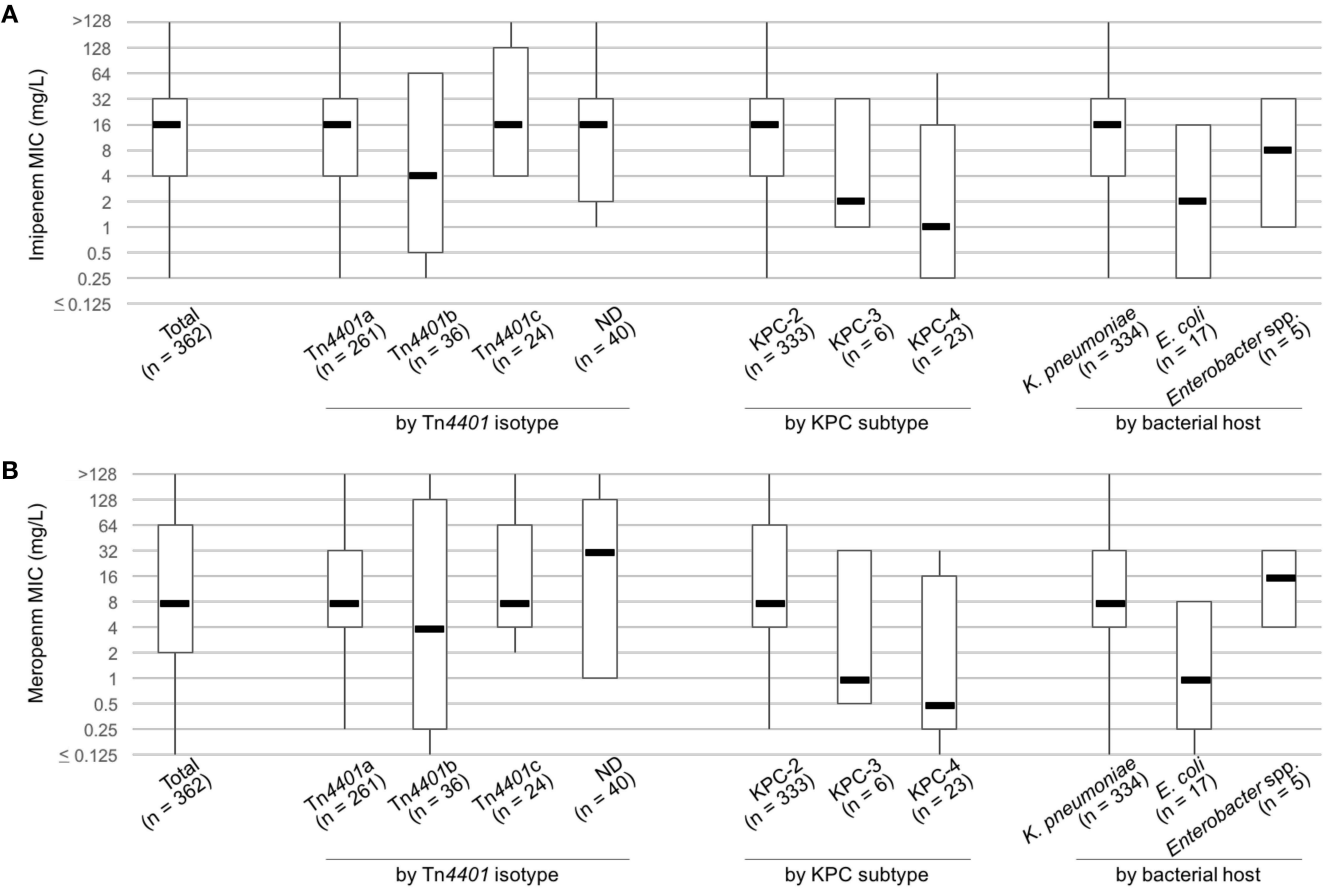
MICs of imipenem **(A)** and meropenem **(B)** in KPC producers. Boxplots present MIC_50_, MIC_10_, and MIC_90_ values, with whiskers showing the maximum and minimum MICs of the group.

Overall MIC_50_s of imipenem and meropenem in KPC producers were 16 mg/L and 8 mg/L, respectively (Figure 2), and the values met the three predominant factors in the KPC populations: the host bacterial species (*K. pneumoniae*), the resistance gene (*bla*_KPC-2_), and the transposon encoding the gene (Tn*4401a*). The KPC-producing *K. pneumoniae* had three-dilution higher MIC_50_s of the two carbapenems than *E. coli* and KPC-2 producers had three- and four-dilution higher MIC_50_ values than to the KPC-3 and KPC-4 producers, respectively. The KPC producers carrying Tn*4401a* had one- or two-dilution higher MIC_50_ values of the carbapenemase than KPC producers carrying Tn*4401b*, whereas those carrying Tn*4401c* had the same MIC_50_ values as KPC producers carrying Tn*4401a*. The ND transposon encoding *bla*_KPC-2_ (*n* = 40) had the highest MIC_50_ of meropenem 32 mg/L that was two- to three-dilution higher than the three isotypes of Tn*4401*, whereas that of imipenem looked alike.

### Cross- and co-resistance to other drugs

As a result of cross resistance from producing KPC, all or most of the 362 KPC producers were non-susceptible to aztreonam (100%), cefotaxime (100%), ceftazidime (*n* = 359, 99.2%), and cefoxitin (*n* = 325, 89.8%) (Table 2).

**Table 2:** Resistance phenotypes of KPC producers.

	**Total**	**Number (%) of non-susceptible strains to**								
		**Aztreonam**	**Cefotaxime**	**Ceftazidime**	**Cefoxitin**	**Gentamicin**	**Amikacin**	**Ciprofloxacin**	**Tigecycline**	**Colistin**
KPC-2	334	334 (100)	334 (100)	332 (99.4)	305 (91.3)	203 (60.8)	44 (13.2)	323 (96.7)	77 (23.1)	8 (2.4)
Tn*4401*a	261	261 (100)	261 (100)	259 (99.2)	235 (90.0)	160 (61.3)	17 (6.5)	250 (95.8)	72 (27.6)	4 (1.5)
Tn*4401*b	8	8 (100)	8 (100)	8 (100)	8 (100)	8 (100)	5 (62.5)	8 (100)	0 (0.0)	1 (12.5)
Tn*4401*c	24	24 (100)	24 (100)	24 (100)	22 (91.7)	12 (50.0)	2 (8.3)	24 (100)	4 (16.7)	1 (4.2)
ND	41	41 (100)	41 (100)	41 (100)	40 (97.6)	23 (56.1)	20 (48.8)	41 (100)	1 (2, 0.4)	2 (4.9)
KPC-3	6	6 (100)	6 (100)	5 (83.3)	6 (100)	2 (33.3)	0 (0.0)	6 (100)	0 (0.0)	0 (0.0)
Tn*4401*b	6	6 (100)	6 (100)	5 (83.3)	6 (100)	2 (33.3)	0 (0.0)	6 (100)	0 (0.0)	0 (0.0)
KPC-4	22	22 (100)	22 (100)	22 (100)	14 (63.6)	15 (68.2)	2 (9.1)	19 (86.4)	3 (13.6)	0 (0.0)
Tn*4401*b	22	22 (100)	22 (100)	22 (100)	14 (63.6)	15 (68.2)	2 (9.1)	19 (86.4)	3 (13.6)	0 (0.0)
Total	362	362 (100)	362 (100)	359 (99.2)	325 (89.8)	220 (60.8)	46 (12.7)	348 (96.1)	80 (22.1)	8 (2.2)

A total of 192 KPC producers (53.0%) produced at least one CTX-M-type ESBL (Table 3): 46.7% (169/362) CTX-M type 1 and 8.0% (29/362) CTX-M type 9. Of note, the *bla*_CTX−M_ gene was often possessed by KPC-2 (183/334, 54.8%) and KPC-4 (9/22, 40.9%) producers, whereas none were identified in KPC-3 producers. Plasmid-mediated AmpC was rarely co-produced (13/362, 3.4%) by the KPC producers: DHA (*n* = 9), ACT (*n* = 1), or CMY (*n* = 1) production in KPC-2 producers and ACC (*n* = 2) production in KPC-4 producers.

**Table 3:** Co-producing drug CTX-M-type extended-spectrum beta-lactamase.

	**Total**	**CTX-M-type extended-spectrum beta-lactamase**		
		**Type 1**	**Type 9**	**Both types 1 and 9**
KPC-2	334	156 (46.7)	21 (6.3)	6 (1.8)
Tn*4401a*	261	136 (52.1)	9 (3.4)	6 (1.8)
Tn*4401b*	8	2 (25.0)	1 (12.5)	0 (0.0)
Tn*4401c*	24	5 (20.8)	0 (0.0)	0 (0.0)
ND	41	13 (31.7)	11 (26.8)	0 (0.0)
KPC-3	6	0 (0.0)	0 (0.0)	0 (0.0)
Tn*4401b*	6	0 (0.0)	0 (0.0)	0 (0.0)
KPC-4	22	7 (31.8)	2 (9.1)	0 (0.0)
Tn*4401b*	22	7 (31.8)	2 (9.1)	0 (0.0)
Total	362	163 (45.0)	23 (6.4)	6 (1.7)

Among the 362 KPC producers, 220 (60.8%) and 46 (12.7%) were resistant to gentamicin and amikacin, respectively, but only few KPC-2 producers possessed the 16S ribosomal methyltransferase *armA* (1/362, 0.3%) and *rmtB* (22/362, 6.1%) genes.

A total of 348 isolates (96.7%) were non-susceptible to ciprofloxacin and 80 (22.1%) and eight (2.2%) isolates were non-susceptible to tigecycline and colistin, respectively. None of the eight colistin-resistant strains possessed the *mcr* gene.

## Discussion

KPC enzymes are able to hydrolyze penicillins, cephalosporins, monobactams, carbapenems, and even β-lactamase inhibitors. Therefore, the wide spread of the producers is one of the worrisome global issues encountering antimicrobial resistance. In accordance of occasional reports (Rhee et al., 2010; Hong et al., 2013; Yoo et al., 2013) and data from NLSS (unpublished data), only infrequent occurrence of the KPC producers were identified in South Korea until 2013. However, after a sudden expansion of KPC producers in 2014, rapid nation-wide spread supported by single-hospital outbreaks (Kim et al., 2017), frequently associated with inter-hospital or inter-regional spread (Jeong et al., 2016) led the endemic stage of KPC in 2015. Actually, the KPC producer in 2013 has never been identified again and the four *K. pneumoniae* subclones, ST307/Tn*4401a*[*bla*_KPC-2_], ST307/Tn*4401b*[*bla*_KPC-4_], ST392/Tn*4401b*[*bla*_KPC-4_], and ST11/ND[*bla*_KPC-2_] in 2014 triggered the inter-regional dissemination of KPC producers.

In agreement with the Healthcare Delivery System in South Korea, convalescent hospitals are in charge post-acute care and general hospitals are responsible for acute care, leading a habitual inter-hospital patient transfer. In general, the infection control system in convalescent hospitals is poorly operated compared to that in general hospitals and their role as a reservoir of CPE has been highly suspected. Indeed, the core KPC-producing subclone *K. pneumoniae* ST307/Tn*4401a*[*bla*_KPC-2_] was identified in all three types of healthcare facilities: 153 of the 362 KPC producers from general hospitals, one of the three isolates from the two convalescent hospitals and one of the two isolates from clinics. It suggested that the KPC has disseminated during the inter-hospital patient transfer and expanded up to the community level. Further study with more isolates from the two types of healthcare facility is needed.

The well-known KPC-producing clone in a global level is *K. pneumoniae* ST258 (Pitout et al., 2015; Sheppard et al., 2016), however, it was not the case in South Korea. The foremost South Korean KPC producer was *K. pneumoniae* ST307 clone and the ST11 was followed by presenting half the prevalence of ST307. Indeed, the ST258 (*gapA*-*infB*-*mdh*-*pgi*-*phoE*-*rpoB*-*tonB*, 3-3-1-1-1-1-79) is a deduced variant of ST11 (3-3-1-1-1-1-43) manufactured by multiple events of a genetic rearrangement (Breurec et al., 2013). *K. pneumoniae* ST307, which has gained attention as a potentially high-risk KPC producer, has been a notorious clone producing CTX-M-15 (Villa et al., 2017) and the clone co-producing KPC and CTX-M-15 was recently identified in Italy (Geraci et al., 2015). The South Korean ST307 clone are assumed to be close to the Italian clone in an evidence of 71.9% KPC-producing *K. pneumoniae* ST307 co-produced CTX-M enzyme. The second-most disseminated *K. pneumoniae* ST11 is one of the main KPC-producing clones in China, and it frequently co-harbors the *rmtB* genes (Cheng et al., 2016). The 22 RmtB-co-producers were all *K. pneumoniae* ST11 representing 28.7% (22/77) the clone.

KPC-2 and KPC-3 are the prevailing global subtypes of KPC, with regional variation (Munoz-Price et al., 2013). Among the South Korean KPC producers, KPC-4 was the second most prevalent subtype, although the number of KPC-4 producers was only one fifteenth that of the KPC-2 producers. Since the bacterial host species varied, the Tn*4401b*-associated plasmid (along with sporadic occurrence) was likely responsible for its observed dissemination. KPC-3, which was produced by fewer bacterial hosts, was limited to one district. KPC-2, KPC-3, and KPC-4 each confer a similar level of carbapenem resistance (Mehta et al., 2015). However, among the KPC producers studied, KPC-2 had the highest MIC_50_s for carbapenems, while KPC-3 and KPC-4 had 3- and 4-dilution-reduced MIC_50_s, respectively (Figure 2). It is likely that multiple factors (such as small relative group size, or low gene expression in the Tn*4401b* isotype) accounted for this unexpected result.

Although group size varied (334 vs. 17), the KPC-producing *K. pneumoniae* isolates had 3-dilution higher MIC_50_s for carbapenems compared to the KPC-producing *E. coli* isolates (Figure 2), pronouncing the importance of bacterial host background for revealing resistance. As well, the Tn*4401* isotypes, differing the promoter of the *bla*_KPC_ gene, is closely associated with the level of gene expression resulting in varied carbapenem susceptibility (Naas et al., 2012; Cheruvanky et al., 2017). For example, Tn*4401a* and Tn*4401d* possessing two promoters upstream from the gene confer high carbapenem resistance whereas Tn*4401c* and Tn*4401e*, with one promoter, confer low levels of resistance and the Tn*4401b*, with three promoters, confers moderate resistance (Naas et al., 2012; Cheruvanky et al., 2017). Three isotypes of the transposon that are Tn*4401a*, Tn*4401b*, and Tn*4401c* were identified in our study. Tn*4401a* had the highest MIC_50_s for carbapenems and the Tn*4401b* exhibited the lowest values. Tn*4401c* of similar MIC_50_ values to those of Tn*4401a* could be explained by the bacterial host, as *K. pneumoniae* likely, somehow, has the most efficient KPC production. The relatively high meropenem MIC_50_ by ND transposon encoding the *bla*_KPC-2_ compared to the other isotypes of Tn*4401* needed to be further studied.

Though the global spread of KPC-producing *Enterobacteriaceae* is becoming more common, local epidemiology patterns need to be actively assessed. An awareness of the details of regional epidemiology is fundamental for understanding specific situations. Most endemic cases have occurred via the clonal dissemination of notorious KPC-producing clones, and the South Korean situation was no exception. The disseminated *K. pneumoniae* clones in South Korea have been linked to the Italian ST307 and Chinese ST11 strains, confirming the importance of international and intercontinental travel for clonal spread. There remains a paucity of antimicrobial options to treat pathogens capable of KPC production, which raises mortality rates. Only a few new drug candidates are in development at present. Combination therapy using both β-lactams and β-lactamase inhibitors that are stable in the presence of KPC enzymes (such as avibactam) is available in other parts of the world, but is not yet accessible in South Korea. Because South Korea is facing endemic dissemination, the situation is critical. Only systematic surveillance will empower researchers to control and prevent the spread of KPC producers.

## Ethics statement

The research, which has no involvement of human subject but the clinical isolates, does meet the exempt category without approval from Ethics Committee on Human Research of the Health Ministry in South Korea and the study design has not been reviewed by the committee.

## Author contributions

SJ and E-JY: contributed to idea conception, designed the study, conducted the experiments, interpreted the data, and wrote the manuscript; JK, DK, HL, JY, and KL: conducted the experiments and interpreted the data.

### Conflict of interest statement

The authors declare that the research was conducted in the absence of any commercial or financial relationships that could be construed as a potential conflict of interest.
