# Supplementary material for: Klebsiella pneumoniae Carbapenemase Producers in South Korea between 2013 and 2015
Source: Front Microbiol. 2018 Jan 25;9:56. doi: 10.3389/fmicb.2018.00056 (PMC5788937; doi:10.3389/fmicb.2018.00056)
Supplement: Supplementary file 1 [file Table1.PDF]

Supplementary Table. Primers used in the study

Name	Sequence (5'-3')	Purpose	Specific for Tn*4401* isotype:
For bla_{KPC} subtyping			
*bla*KPC-F	ATGTCACTGTATCGCCGTCT	PCR/Sequencing	-
*bla*KPC-R	TTTTCAGAGCCTTACTGCCC	PCR/Sequencing	-
For Tn*4401* isotyping			
abcde—396-377_F_495	ACCCGAATGATCCAGGTGGG	PCR	a / b / c / d / e
abcd_—275-256_F_374	CTTGGCCAGGACTTCCTGAG	PCR	a / b / c / d
_b___—266-147_F_265	GGTGCCAGGGACTTACCAAC	PCR	b
_b_d_—114-95_F_213	GTTACAGCCTCTGGAGAGGG	PCR	b / d
ab_d_-69—50_F_168	CCAGCTGTAGCGGCCTGATT	PCR	a / b / d
kpc_uni-80-99_R	GAATGGTTCCGCGACGAGGT	PCR/Sequencing	a / b / c / d / e
